# Trends in the prevalence of pediatric lower urinary tract symptoms in a national claims database of privately insured patients, 2007-2016

**DOI:** 10.3389/fruro.2025.1422897

**Published:** 2025-04-16

**Authors:** Raphael James Brosula, Pranaya Venkatapuram, Abby L. Chen, Chiyuan A. Zhang, Kathleen M. Kan

**Affiliations:** Department of Urology, Stanford University School of Medicine, Palo Alto, CA, United States

**Keywords:** lower urinary tract symptoms, administrative claims, healthcare, retrospective studies, pediatrics, urology

## Abstract

**Background:**

Pediatric lower urinary tract symptoms (LUTS) impact a significant number of children and families worldwide. Estimated prevalences rely on small cross-sectional studies, leading to inconsistent estimates. This study aims to characterize demographic and temporal trends in LUTS prevalence within a national claims database of privately insured individuals in the United States.

**Methods:**

We conducted a retrospective cohort study by reviewing the Merative™ MarketScan^®^ Outpatient Research Database v2.0 between 2007-2016. Patients with neurogenic bladder, renal transplant, structural urologic disease, and concurrent urinary tract infection were excluded. Yearly trends were reviewed across age, sex, geographic region, and clinical comorbidities such as attention-deficit/hyperactivity disorder (ADHD) and constipation. Yearly frequency of diagnostic codes was calculated to characterize LUTS diagnostic coding practices.

**Results:**

We identified 1,625,538 patients aged 5-18 years with LUTS, representing 6% of the total population at risk, with a median age of 8.0 years. More patients in the cohort were female (66.1%), between 5-10 years old (57.9%), and resided in the Southern US (38.5%). The yearly prevalence of LUTS significantly increased from 1.8% to 2.1% yearly, and saw significant increases in females, 15-18 year old patients, and across several geographic regions. Comorbid constipation and ADHD within LUTS patients also significantly increased. Diagnostic coding practices remained stable.

**Conclusions:**

Families of patients with LUTS are increasingly seeking medical care for their condition. These results exceed similar estimates from previous longitudinal studies and can inform population-level intervention strategies. Further studies should investigate the impact of LUTS on healthcare resource utilization, including in non-privately insured populations.

## 1 Introduction

Pediatric lower urinary tract symptoms (LUTS) are commonly seen among children in the United States, affecting around 17% of school-aged children, the majority between the ages of 5-10 years old who present with daytime and nighttime incontinence, urgency, frequency, and dysuria (1–4). Factors associated with LUTS presentation include gender, family history of incontinence, and clinical comorbidities such as constipation and attention deficit/hyperactivity disorder (ADHD) (4–9). The sequelae of LUTS includes a decrease in quality of life and self-esteem, increased family stress, recurrent urinary tract infection (UTI), and an uncalculated burden of missed school and work days (10–13). Further understanding the population-level prevalence of associated factors of pediatric LUTS may help to address the needs of children and families seeking care for this condition.

The prevalence of families with LUTS seeking medical care has not been recently well-defined. In 2012, the *Urologic Diseases in America* report estimated that approximately 1,000 per 100,000 children in the United States ≤10 years old required outpatient care for a pediatric urinary incontinence diagnosis (14). Existing studies based on cross-sectional surveys differ in study techniques, questionnaires, and criteria for LUTS, limiting the direct comparison of findings. Further characterizing this population in terms of their demographics and clinical comorbidities over time is necessary to understand disease burden and longitudinal risk as well as to inform the scope, feasibility, and effectiveness of population-level treatment strategies for pediatric LUTS.

The Merative™ MarketScan^®^ Outpatient Research Database is a US database containing individual level, de-identified healthcare claims information integrating data from commercial medical insurance claims of privately insured patients. While results are limited due to the differences in population characteristics in a commercially insured versus uninsured patient group, this dataset represents a unique opportunity to characterize the trends in prevalence within a LUTS cohort at a national level, with the potential to be utilized for future healthcare resource utilization studies. Using claims data, we aim to define the overall and yearly prevalence of LUTS in a pediatric population ages 5-18 years old as well as the yearly prevalence of demographic variables and clinical comorbidities within this cohort.

## 2 Materials and methods

### 2.1 Data source

We analyzed data derived from the Merative™ MarketScan^®^ Outpatient Research Database v2.0 between the years 2007 to 2016. The de-identified data include enrollment data from large employers and health plans across the United States providing healthcare coverage for more than 153 million employees, their spouses, and dependents in total. Enrollment, demographic, and claims data are available. This includes services provided in a doctor’s office, hospital-based outpatient facility and/or emergency department. Diagnoses were coded using the International Classification of Diseases, Ninth Revision (ICD-9) and Tenth Revision (ICD-10). Procedures were coded using Current Procedural Terminology (CPT).

### 2.2 Ethics

Research on de-identified data hosted by the Stanford Center for Population Health Sciences is conducted under protocol 40974. This protocol includes a waiver of consent, a waiver assent and a waiver of HIPAA authorization.

### 2.3 Study population

We conducted a retrospective population-based cohort study. Our cohort included all patients aged 5-18 years old with LUTS (**Appendix 1**; see **Supplementary Material**). A patient was considered to have LUTS if they had at least 1 LUTS diagnosis code at any time between 2007-2016 within an outpatient visit claim. To prevent confounding of LUTS prevalence measurements, we excluded patients with an ICD-9/ICD-10 diagnosis code or CPT surgical code related to neurogenic bladder, renal transplant, and structural urologic diseases - hypospadias, vesicoureteral reflux, posterior urethral valves, urethral stricture disease, and ureterocele - that increase risk for LUTS. To avoid further confounding, we excluded patient encounters with a concurrent urinary tract infection (UTI) code in the same claim (**Appendix 1**; see **Supplementary Material**). Clinically, LUTS cases often present without a concurrent UTI diagnosis, and lack of laboratory data in the database precludes further confirmation of UTI cases in our cohort.

### 2.4 Statistical analysis

The primary outcome was prevalence of LUTS per year between 2007 to 2016. Secondary outcomes were the stratification of LUTS prevalence via demographic variables and clinical comorbidities. These stratified prevalence rates were calculated yearly and within the cohort. Demographic variables analyzed include age, sex, and geographic region (North Central, Northeast, South, West). Clinical comorbidities analyzed include constipation and ADHD. A patient was defined to have constipation if they had at least one constipation-related diagnosis code at any time in their record. A patient was defined to have ADHD if they had at least two ADHD-related diagnoses codes within their record. Requiring multiple codes addresses the frequent use of ADHD-related diagnosis codes during screening encounters and is similar to prior methods identifying ADHD within claims databases (15, 16).

The overall prevalence of LUTS was calculated as the proportion of unique LUTS patients within the total population at risk. The yearly prevalence of LUTS was calculated as the proportion of LUTS patients within the total population at risk per one-year period. We conducted a descriptive analysis of the cohort’s demographic characteristics and clinical comorbidities at the time of the first diagnosis.

Additionally, trends over time for the primary and secondary outcomes were characterized using a Mann-Kendall trend test with a statistically significant trend at p < 0.05, adjusting with the Bonferroni correction for multiple hypotheses. Trends in diagnostic coding practices were evaluated to address potential limitations to using a claims database. Yearly frequency of the top four diagnoses was calculated from 2007-2016 as a proportion of unique patients and unique encounters with the corresponding diagnoses code.

## 3 Results

We identified a total population at risk of 26,977,105. After applying exclusion criteria, we identified a total LUTS cohort with 1,625,538 patients, representing a 6.03% average prevalence in the dataset. The median age of LUTS patients was 8.0 years. Within the cohort, more patients were female (66.1%), in the 5-10 age group (57.9%), and from the South (38.5%) (**Table 1**). 5.4% of the patients within the cohort had comorbid constipation, and 1.8% had ADHD.

**Table 1:** Descriptive statistics and prevalence trends by demographics and clinical comorbidities within the LUTS cohort.

	n (%)	Prevalence Trend (p-value)
Overall	1,625,538	**Increasing (p = 0.007)**
Sex		
Male	551,607 (33.9)	**No trend (p = 0.012)**
Female	1,073,931 (66.1)	**Increasing (p = 0.004)**
Age Group at First Diagnosis		
5-10	941,856 (57.9)	No trend (p = 0.721)
11-14	276,857 (17.0)	No trend (p = 0.074)
15-18	408,625 (25.0)	**Increasing (p = 0.002)**
Region		
North Central	408,843 (25.2)	**No trend (p = 0.032)**
Northeast	285,496 (17.6)	**Increasing (p = 0.007)**
South	626,142 (38.5)	**Increasing (p = 0.007)**
West	256,883 (15.8)	**Increasing (p < 0.001)**
Unknown	48,174 (3.0)	No trend (p = 0.283)
Comorbidity		
ADHD	28,516 (1.8)	**Increasing (p < 0.001)**
Constipation	87,318 (5.4)	**Increasing (p < 0.001)**

Bold values indicate statistically significant monotonic trends.

**Figure 1:**
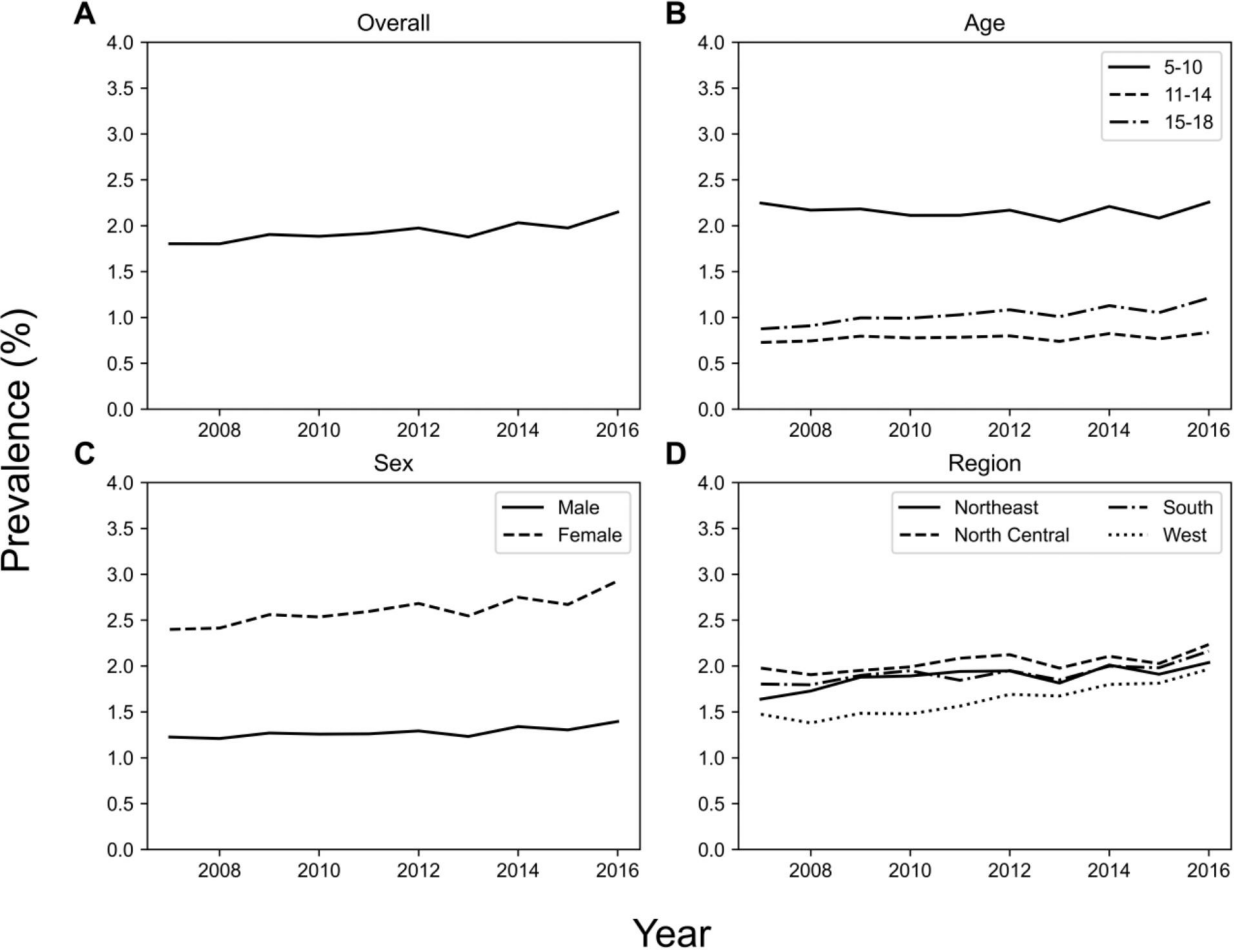
Across several demographics, LUTS prevalence increased from 2007 to 2016. The plots show **(A)** overall prevalence, and prevalence stratified by **(B)** age group, **(C)** sex, and **(D)** geographic region.

Yearly prevalence of LUTS between 2007 and 2016 are shown in **Figure 1**. Overall LUTS prevalence increased (p = 0.007) from 1.8% in 2007 to 2.2% in 2016. LUTS prevalence stratified by age group, sex, and region are also shown in **Figure 1**. LUTS prevalence increased in the 15-18 years old age group (p = 0.002) from 0.9% in 2007 to 1.2% in 2016, but did not reach significance in the 5-10 and 11-14 year old age groups. LUTS prevalence increased significantly among female patients (p = 0.004) and was consistently higher than males, increasing from 2.4% in 2007 to 2.9% in 2016 compared to 1.2% in 2007 to 1.4% in 2016 for males. Yearly LUTS prevalence increased in the South, Northeast, and West regions (**Figure 1**).

**Figure 2:**
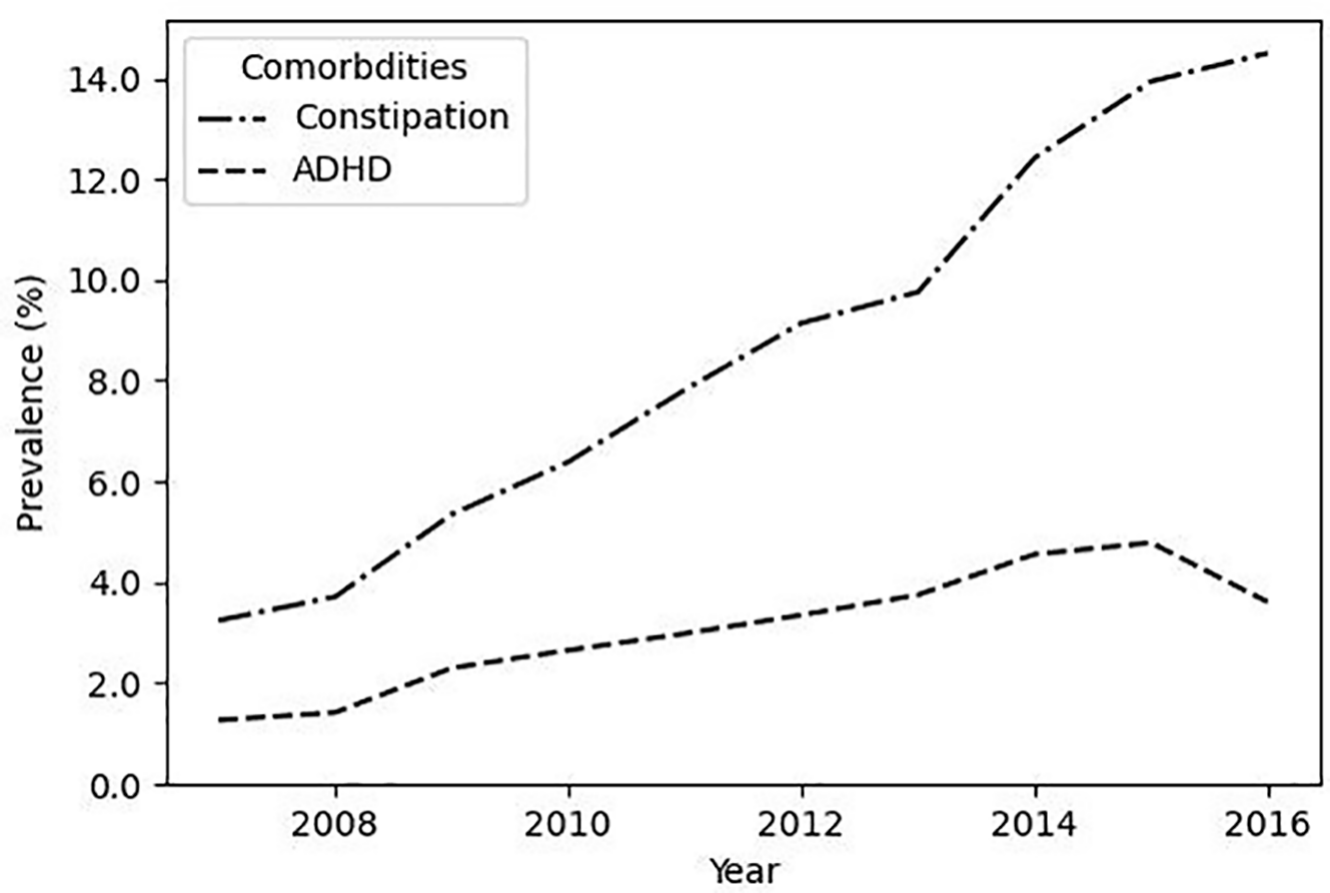
The prevalence of co-morbid constipation increases in LUTS patients. Plotted are the prevalence of co-morbid ADHD and co-morbid constipation within the LUTS cohort.

Yearly prevalence of comorbidities is shown in **Figure 2**. The prevalence of constipation within the LUTS cohort increased from 3.2% in 2007 to a peak of 14.5% in 2016 (p < 0.001). The prevalence of comorbid ADHD also significantly increased (p < 0.001), ranging from 1.3% in 2007 to 3.6% in 2016, and peaking at 4.8% in 2015.

**Figure 3:**
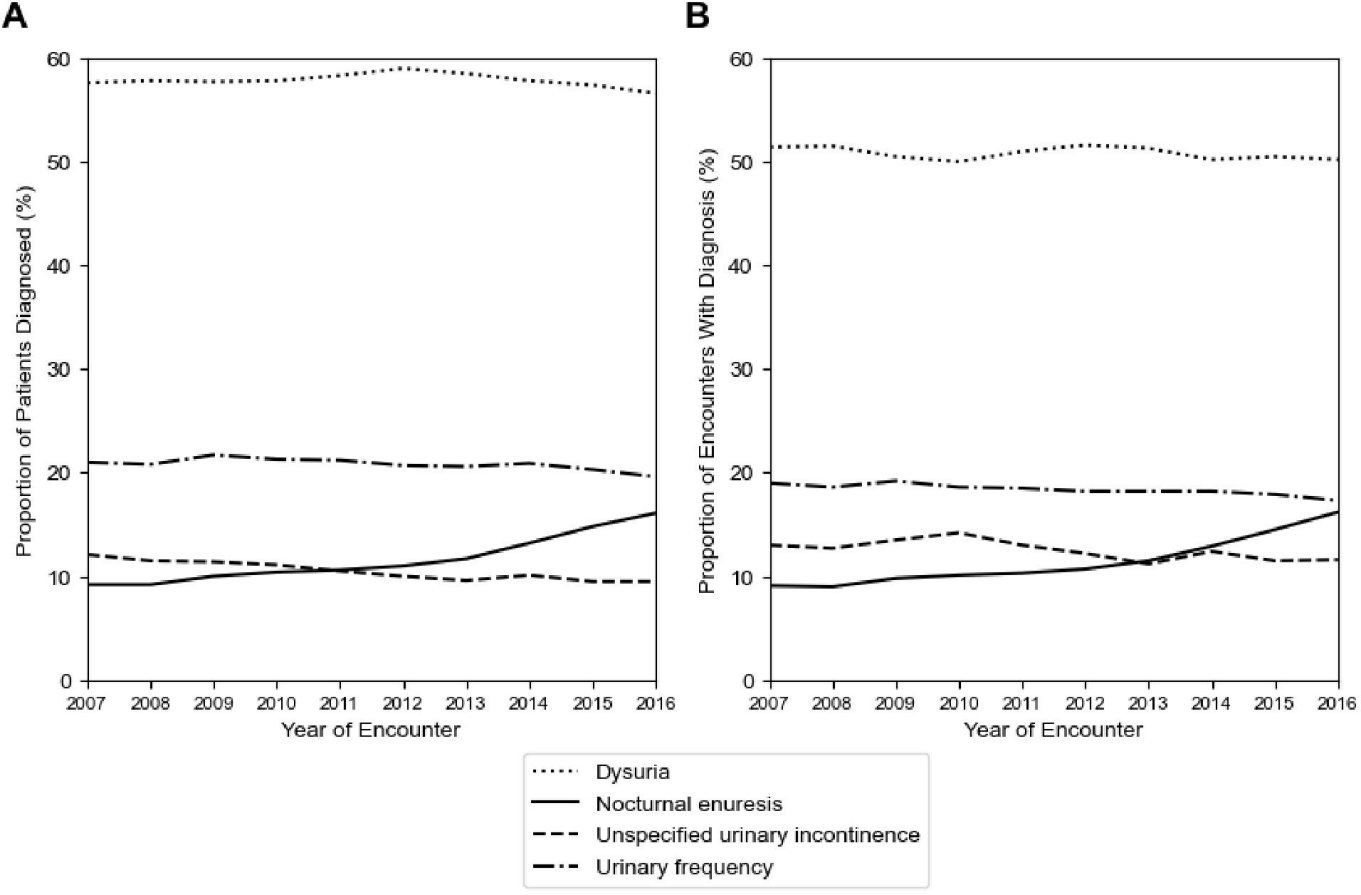
Diagnostic coding practices for LUTS remain stable across the study period. Plotted are **(A)** the yearly proportion of unique patients with a specific LUTS diagnosis, and **(B)** the yearly proportion of unique encounters with a specific LUTS diagnosis.

To investigate LUTS diagnostic coding practices, the top 10 LUTS ICD-9 codes were analyzed by the number of unique patients and encounters (**Table 2**). The top four LUTS diagnoses across both the ICD-9 and ICD–10 periods were the same: dysuria (ICD-9: 788.1, ICD-10: R30.0), urinary frequency/frequency of micturition (ICD-9: 788.41, ICD-10: R35.0), nocturnal enuresis (ICD-9: 788.36, ICD-10: N39.44), and unspecified urinary incontinence (ICD-9: 788.30, R32). Among all patients, 61.1%, 23.1%, 11.1%, and 10.8% had the respective LUTS diagnoses. Similarly, 50.8%, 18.4%, 11.2% and 12.6% of all encounters had the respective LUTS diagnoses. Overall, relative frequency of these diagnostic codes remained largely similar between the two ICD periods except for a shift in increasing nocturnal enuresis as opposed to unspecific urinary incontinence late in the ICD-10 period (**Figure 3**).

**Table 2:** Top 10 ICD-9 LUTS Diagnosis Codes by number and proportion of unique patients and unique encounters with this diagnosis code.

ICD-9 Code	Diagnosis	Number of Unique Patients (%)	Number of Unique Encounters (%)
788.1	Dysuria	913,866 (61.0)	1,191,321 (50.9)
788.41	Urinary frequency	346,297 (23.1)	433,098 (18.5)
788.30	Urinary incontinence, unspecified	162,167 (10.8)	297,401 (12.7)
788.36	Nocturnal enuresis	160,758 (10.7)	250,538 (10.7)
307.6	Enuresis	66,473 (4.4)	118,157 (5.0)
788.63	Urgency of urination	45,292 (3.0)	55,044 (2.4)
599.9	Unspecified disorder of urethra and urinary tract	32,458 (2.2)	39,042 (1.7)
788.69	Other abnormality of urination	23,579 (1.6)	28,669 (1.2)
788.31	Urge incontinence	16,822 (1.1)	26,494 (1.1)
788.21	Incomplete bladder emptying	10,283 (0.7)	14,853 (0.6)

## 4 Discussion

Within the Merative™ MarketScan^®^ Research Database, we find that cumulatively, about 6% of all patients sought medical care for LUTS. Yearly prevalence increased from 1.8% in 2007 to 2.2% in 2016. Most patients were younger, female, and resided in the Southern US. LUTS prevalence increased in female patients, in 15-18 year olds, and in the South, Northeast, and West geographic regions. The prevalence of co-morbid constipation also increased.

Cross-sectional studies estimate that the prevalence of daytime incontinence (DUI), urge incontinence (UI), and urgency/frequency approach 17% (1–3). Additional estimates vary widely due to differences in measuring one presentation of LUTS versus all LUTS, varying age group cutoffs, and the inconsistent use of validated survey instruments to measure disease burden (3, 8, 17–19). In our study, prevalence increased between 1.8% to 2.2%. This number is appropriately lower than community data and reflects a subset of privately insured patients who interact with the healthcare system. In one survey, only 16% and 31.9% of families affected by DUI and nocturnal enuresis (NE) sought any kind of medical care, respectively (9, 20).

Treatment-seeking behaviors may be influenced by factors such as access to care, symptom frequency/severity, or community norms. Compared to previously reported analyses, our findings demonstrate a nearly doubled rate of overall healthcare utilization. The *Urologic Diseases in America* report approximates that 1,000 of every 100,000 families with pediatric incontinence seek outpatient care (14). However, there are some key differences between the two claims-based studies. Our study uses a different database and includes non-incontinence codes relevant to LUTS, such as dysuria and urinary frequency that represent a large proportion of coded visits.

LUTS prevalence increased significantly for girls and compared to boys, prevalence was consistently twice as high. Though some studies have found higher rates of LUTS in girls (8, 9, 17, 19), another study found no statistically significant relation between LUTS and sex (7). This may be because different types of LUTS have different distributions across age and sex. For example, girls demonstrate higher rates of DUI as they age; in contrast, boys show greater rates of NE (21). UTI is more commonly associated with girls and can be associated with LUTS presentations; however, we excluded these UTI diagnoses in our analysis (7).

While the 5-10 year age group was the most frequently diagnosed with LUTS, 15-18 year olds made up 25% of the cohort, and were the only age group where disease prevalence significantly increased over time. Of note, 5% of children at 9.5 years old experience daytime incontinence, suggesting that there is a large group that does not experience symptom resolution (22). Similarly, outpatient office visits for LUTS decreased to 440 per 100,000 in 11-17 year olds, but again, reflect a trend within a subset of older patients who may still seek care (14). Driving factors may include new or chronic symptoms, increased symptom severity, and increased preference for independence in social activities.

Our study found that overall prevalence of co-morbid constipation was 5.4%. Constipation affects 0.7% to 29.6% of children (23). Children with constipation are 6.8 times more likely to have LUTS (8, 25). Thirty-five to thirty-eight percent of children with LUTS have constipation (8, 24). The relationship between clinical presentations may be due to underlying anatomic proximity and overall pelvic floor mechanics (26). The prevalence of co-morbid constipation increased significantly from 3.2% in 2007 to 14.5% in 2016. This change may be due to broader community-based factors such as diet and healthy food access. The highest proportion of children with LUTS in our cohort reside in the Southern US, where higher rates of constipation have been previously observed (27). LUTS diagnoses increased across three geographic areas in this analysis, though the role of this variable cannot be fully captured in claims data alone.

Approximately 42% of children with LUTS also present with ADHD and these children are 2-4 times more likely to experience LUTS compared to children without ADHD (4, 6, 28). The present study finds that 1.8% of children with LUTS also presented with ADHD, and that it significantly increased from 1.3% in 2007 to 3.6% in 2016. These two conditions may be related due to increased difficulties with externalizing problems and oppositional behavior that have been noted in children with DUI (29). Overall, characterizing LUTS comorbidities such as constipation and ADHD is important to establish correct clinical diagnosis, provide appropriate multidisciplinary care, and develop tailored outreach and education programs to address the specific needs of these children.

Our analysis of ICD code frequency reveals that dysuria is the most prominent LUTS-related diagnosis within our database, followed by urinary frequency, nocturnal enuresis, and unspecified urinary incontinence. These findings persist even after the transition between ICD-9 and ICD-10 systems, providing a robust picture of which LUTS people seek medical care for and how providers code for different types of LUTS. Because the relative frequency of these LUTS diagnoses did not increase over time, diagnostic coding patterns are unlikely to be driving the changes observed in LUTS prevalence.

Though the Merative™ MarketScan^®^ Research Databases cover millions of patients across the United States, this study does have limitations. The Merative™ MarketScan^®^ Research Database represents a privately insured population and it lacks data related to race and socioeconomic status. This limits the analysis of risk factors and burden driven by social determinants of health that impact LUTS patients. Further research is needed to contextualize these trends across different social determinants of health and in different populations not captured within the dataset, such as those who are not privately insured. Claims databases are also subject to missing/inaccurate data and selection biases dependent on case definitions, which may include length of enrollment (30). Our study does not account for a specific length of enrollment to ensure that the maximal cohort and total population at risk are accounted for within the prevalence estimate. Though the use of clinically assessed ICD-9/ICD-10 codes for LUTS standardizes the case definition, the sensitivity and specificity of these codes and the accuracy of coding has not been investigated. Our cohort excluded children with renal transplant, neurologic bladder, structural urologic diseases, and concurrent UTI to prevent confounding the measurement of LUTS prevalence. However, the influence of additional conditions such as developmental delay and the role of UTI were not investigated.

Other limitations of this study include 1) those associated with using a private claims database to estimate prevalence within the population overall such as providers’ coding practices and fluctuations in enrollment and access to coverage and 2) the availability of older data only—due to institutional policies on data access—which may not fully reflect current trends.

Further investigation into healthcare resource utilization from claims data allows these programs to target areas of improvement to reduce healthcare spending and prompt investment into preventative programs.

In conclusion, we found that prevalence of pediatric LUTS increased 1.8% to 2.1% between 2007 to 2016 among a population of privately insured patients in the MarketScan^®^ Research Database, revealing that families are increasingly seeking care to manage the conditions of children with pediatric LUTS. This figure is two times that of previous estimates in some of the literature, pointing to a consistent burden within the healthcare system. We also characterize increases in LUTS prevalence within female patients, older children, as well as increases in comorbid ADHD and constipation. Longitudinal study methods can further improve our current understanding of these clinical and demographic risk factors for LUTS to help inform effective treatment and prevention strategies, while prompting investment into appropriate prevention and intervention programs.

## Data Availability

The data that support the findings of this study are available from Merative MarketScan^®^ Research Databases through the Stanford Center for Population Health Sciences, but restrictions apply to the availability of these data, which were used under license for the current study, and so are not publicly available. Data is not available from the authors due to restrictions by Merative MarketScan^®^. Requests to access these datasets should be directed to PHS Data Core, phsdatacore@stanford.edu.
