# Supplementary material for: Trends in the prevalence of pediatric lower urinary tract symptoms in a national claims database of privately insured patients, 2007-2016
Source: Front Urol. 2025 Apr 16;5:1422897. doi: 10.3389/fruro.2025.1422897 (PMC12327348; doi:10.3389/fruro.2025.1422897)
Supplement: Supplementary file 1 [file Table1.docx]

**Appendix 1: ICD-9, ICD-10, and CPT codes related to pediatric lower urinary tract symptoms, comorbidities and exclusion criteria**

| **LUTS diagnosis codes** | | |
| --- | --- | --- |
| **Description** | **ICD-9 Code** | **Code Description** |
| LUTS | 307.6 | Enuresis |
|  | 596.0 | Bladder neck obstruction |
|  | 596.51 | Hypertonicity of bladder |
|  | 599.89 | Other specific disorders of urinary tract |
|  | 599.9 | Unspecified disorder of urethra and urinary tract |
|  | 625.6 | Stress incontinence (female) |
|  | 788.1 | Dysuria |
|  | 788.21 | Incomplete bladder emptying |
|  | 788.3 | Urinary incontinence, unspecified |
|  | 788.31 | Urge incontinence |
|  | 788.33 | Mixed incontinence (male/female) |
|  | 788.34 | Incontinence without sensory awareness |
|  | 788.35 | Post-void dribbling |
|  | 788.36 | Nocturnal enuresis |
|  | 788.38 | Overflow incontinence |
|  | 788.39 | Other Urinary Incontinence |
|  | 788.41 | Urinary frequency |
|  | 788.62 | Slowing of urinary stream |
|  | 788.63 | Urgency of urination |
|  | 788.64 | Urinary hesitancy |
|  | 788.65 | Straining on urination |
|  | 788.69 | Other abnormality of urinary stream/urination |
| **Description** | **ICD-10 Code** | **Code Description** |
| LUTS | N32.0 | Bladder neck obstruction |
|  | N32.81 | Overactive bladder |
|  | N39.3 | Stress incontinence (male/female) |
|  | N39.41 | Urge incontinence |
|  | N39.42 | Incontinence without sensory awareness |
|  | N39.43 | Post-void dribbling |
|  | N39.44 | Nocturnal enuresis |
|  | N39.46 | Mixed incontinence (male/female) |
|  | N39.49 | Other specified urinary incontinence |
|  | N39.490 | Overflow incontinence |
|  | N39.492 | Postural (urinary incontinence) |
|  | N39.498 | Other specified urinary incontinence: reflex incontinence, total incontinence |
|  | N39.8 | Other specified disorders of the bladder |
|  | N39.9 | Disorder of urinary system, unspecified |
|  | R30.0 | Dysuria |
|  | R30.9 | Painful micturition, unspecified |
|  | R32 | Unspecified urinary incontinence |
|  | R35.0 | Frequency of micturition |
|  | R39.11 | Hesitancy of micturition |
|  | R39.12 | Poor urinary stream |
|  | R39.14 | Feeling of incomplete bladder emptying |
|  | R39.15 | Urgency of urination |
|  | R39.16 | Straining to void |
|  | R39.19 | Other difficulties with micturition |
|  | R39.191 | Need to immediately revoid |
|  | R39.198 | Other difficulties with micturition |
|  | R39.9 | Unspecified symptoms and signs involving the genitourinary system |
| **Comorbidities in LUTS Cohort** | | |
| **Description** | **ICD-9 Code** | **Code Description** |
| ADHD | 314.00 | Attention deficit disorder of childhood without hyperactivity |
|  | 314.01 | Attention deficit disorder of childhood with hyperactivity |
| Constipation | 307.7 | Encopresis |
|  | 560.32 | Fecal impaction |
|  | 564.0 | Constipation |
|  | 564.01 | Slow transit constipation |
|  | 564.02 | Outlet dysfunction constipation |
|  | 564.09 | Other constipation |
|  | 787.60 | Full incontinence of feces |
|  | **ICD-10 Code** | **Code Description** |
| ADHD | F90.0 | Attention-deficit hyperactivity disorder, predominantly inattentive type |
|  | F90.1 | Attention-deficit hyperactivity disorder, predominantly hyperactive type |
|  | F90.2 | Attention-deficit hyperactivity disorder, combined type |
|  | F90.8 | Attention-deficit hyperactivity disorder, other type |
|  | F90.9 | Attention-deficity hyperactivity disorder, unspecified type |
| Constipation | F98.1 | Encopresis not due to a substance or known physiological condition |
|  | K56.41 | Fecal impaction |
|  | K59.0 | Constipation |
|  | K59.00 | Constipation, unspecified |
|  | K59.01 | Slow transit constipation |
|  | K59.02 | Outlet dysfunction constipation |
|  | K59.03 | Drug induced constipation |
|  | K59.04 | Chronic idiopathic constipation |
|  | K59.09 | Other and unspecified constipation |
|  | R15.9 | Full incontinence of feces |
| **Exclusion Criteria** | | |
| **Description** | **ICD-9 Code** | **Code Description** |
| Renal Transplant | V42.0 | Kidney transplant |
|  | 996.81 | Complication of kidney transplant |
| Neurogenic Bladder | 596.4 | Atony of bladder |
|  | 596.54 | Neurogenic bladder NOS |
|  | 344.61 | Cauda equina syndrome with neurogenic bladder |
| Hypospadias | 58.45 | Repair of hypospadias/epispadias |
|  | 752.61 | Hypospadias (male) |
|  | 753.8 | Other specified anomalies of bladder and urethra |
| Posterior urethral valves | 753.6 | Atresia and stenosis of urethra and bladder neck |
| Vesicoureteral reflux | 593.70 | Vesicoureteral reflux, Unspecified or without reflux nephropathy |
|  | 593.71 | Vesicoureteral reflux, With reflux nephropathy, unilateral |
|  | 593.72 | Vesicoureteral reflux, With reflux nephropathy, bilateral |
|  | 593.73 | Vesicoureteral reflux, With reflux nephropathy NOS |
| Ureterocele | 753.23 | Congenital ureterocele |
| Neurologic disorders | 741.01 | Spina bifida with hydrocephalus, cervical region |
|  | 741.02 | Spina bifida with hydrocephalus, dorsal (thoracic) region |
|  | 741.03 | Spina bifida with hydrocephalus, lumbar region |
|  | 741.00 | Spina bifida with hydrocephalus, unspecified region |
|  | 741.91 | Spina bifida without mention of hydrocephalus, cervical region |
|  | 741.92 | Spina bifida without mention of hydrocephalus, dorsal (thoracic) region |
|  | 741.93 | Spina bifida without mention of hydrocephalus, lumbar region |
|  | 741.9 | Spina bifida without mention of hydrocephalus, unspecified region |
|  | 742.9 | Unspecified anomaly of brain, spinal cord, and nervous system |
|  | 742.59 | Other specified anomalies of spinal cord |
|  | 742.51 | Diastematomyelia |
|  | 742.53 | Hydromyelia |
|  | 343.2 | Congenital quadriplegic |
|  | 343.0 | Congenital diplegia |
|  | 343.1 | Congenital hemiplegic |
|  | 333.71 | Athetoid Cerebral Palsy |
|  | 343.8 | Other specified infantile cerebral palsy |
|  | 343.3 | Congenital monoplegia |
|  | 343.9 | Infantile cerebral palsy, unspecified |
| Urinary tract infection | 599.0 | Urinary Tract infection, site not specified |
|  | 595.0 | Acute cystitis |
|  | 595.9 | Cystitis, unspecified |
|  | 595.3 | Trigonitis |
|  | 590.00 | Chronic polynephritis without lesion of renal medullary |
|  | **ICD-10 Code** | **Code Description** |
| Renal Transplant | Z94.0 | Kidney transplant status |
|  | T86.19 | Other complication of kidney transplant |
|  | T86.13 | Kidney transplant infection |
|  | T86.10 | Unspecified complication of kidney transplant |
|  | T86.12 | Kidney transplant failure |
|  | T86.11 | Kidney transplant rejection |
| Neurogenic Bladder | N31.0 | Uninhibited neuropathic bladder, not elsewhere classified |
|  | N31.1 | Reflex neuropathic bladder, not elsewhere classified |
|  | N31.2 | Flaccid neuropathic bladder, not elsewhere classified |
| Hypospadias | Z87.710 | Personal history of (corrected) hypospadias |
|  | Q54.0 | Hypospadias, balanic |
|  | Q54.1 | Hypospadias, penile |
|  | Q54.2 | Hypospadias, penoscrotal |
|  | Q54.3 | Hypospadias, perineal |
|  | Q54.8 | Other hypospadias |
|  | Q54.9 | Hypospadias, unspecified |
|  | Q64.0 | Epispadias |
| Posterior urethral valves | Q64.2 | Congenital posterior urethral valves |
|  | Q64.39 | Other atresia and stenosis of urethra and bladder neck |
| Vesicoureteral reflux | N13.70 | Vesicoureteral-reflux, unspecified |
|  | N13.71 | Vesicoureteral-reflux without reflux nephropathy |
|  | N13.72 | Vesicoureteral-reflux with reflux nephropathy without hydroureter |
|  | N13.721 | Vesicoureteral-reflux with reflux nephropathy without hydroureter, unilateral |
|  | N13.722 | Vesicoureteral-reflux with reflux nephropathy without hydroureter, bilateral |
|  | N13.729 | Vesicoureteral-reflux with reflux nephropathy without hydroureter, unspecified |
|  | N13.73 | Vesicoureteral-reflux with reflux nephropathy with hydroureter |
|  | N13.731 | Vesicoureteral-reflux with reflux nephropathy with hydroureter, unilateral |
|  | N13.732 | Vesicoureteral-reflux with reflux nephropathy with hydroureter, bilateral |
|  | N13.739 | Vesicoureteral-reflux with reflux nephropathy with hydroureter, unspecified |
| Ureterocele | Q62.31 | Congenital ureterocele, orthotopic |
|  | Q62.32 | Cecoureterocele |
| Neurologic disorders | Q05.0 | Cervical spina bifida with hydrocephalus |
|  | Q05.1 | Thoracic spina bifida with hydrocephalus |
|  | Q05.2 | Lumbar spina bifida with hydrocephalus |
|  | Q05.3 | Sacral spina bifida with hydrocephalus |
|  | Q05.4 | Unspecified spina bifida with hydrocephalus |
|  | Q05.5 | Cervical spina bifida without hydrocephalus |
|  | Q05.6 | Thoracic spina bifida without hydrocephalus |
|  | Q05.7 | Lumbar spina bifida without hydrocephalus |
|  | Q05.8 | Sacral spina bifida without hydrocephalus |
|  | Q05.9 | Spina bifida, unspecified |
|  | Q06.0 | Amyelia |
|  | Q06.1 | Hypoplasia and dysplasia of spinal cord |
|  | Q06.2 | Diastematomyelia |
|  | Q06.3 | Other congenital cauda equina malformations |
|  | Q06.4 | Hydromyelia |
|  | Q06.8 | Other specified congenital malformations of spinal cord |
|  | Q06.9 | Congenital malformation of spinal cord, unspecified |
|  | Q07.0 | Arnold-Chiari syndrome |
|  | Q07.00 | Arnold-Chiari syndrome without spina bifida or hydrocephalus |
|  | Q07.01 | Arnold-Chiari syndrome with spina bifida |
|  | Q07.02 | Arnold-Chiari syndrome with hydrocephalus |
|  | Q07.03 | Arnold-Chiari syndrome with spina bifida and hydrocephalu |
|  | Q07.8 | Other specified congenital malformations of nervous system |
|  | Q07.9 | Congenital malformation of nervous system, unspecified |
|  | Q80.0 | Spastic quadriplegic cerebral palsy |
|  | Q80.1 | Spastic diplegic cerebral palsy |
|  | Q80.2 | Spastic hemiplegic cerebral palsy |
|  | Q80.3 | Athetoid cerebral palsy |
|  | Q80.4 | Ataxic cerebral palsy |
|  | Q80.8 | Other cerebral palsy |
|  | Q80.9 | Cerebral palsy, unspecified |
| Urinary tract infection | N39.0 | Urinary Tract infection, site not specified |
|  | N30.00 | Acute cystitis without hematuria |
|  | N30.01 | Acute cystitis with hematuria |
|  | N30.20 | Other chronic cystitis without hematuria |
|  | N30.21 | Other chronic cystitis with hematuria |
|  | N30.30 | Trigonitis without hematuria |
|  | N30.31 | Trigonitis with hematuria |
|  | N30.80 | Other cystitis without hematuria |
|  | N30.81 | Other cystitis with hematuria |
|  | N30.90 | Cystitis, unspecified without hematuria |
|  | N30.91 | Cystitis, unspecified with hematuria |
|  | N11.0 | Nonobstructive reflux-associated chronic pyelonephritis |
|  | **CPT Code** | **Code Description** |
| Renal Transplant | 50360 | Renal allotransplantation, implantation of graft; without recipient nephrectomy |
|  | 50365 | Renal allotransplantation, implantation of graft; with recipient nephrectomy |
| Hypospadias | 54304 | Plastic operation on penis for correction of chordee or for first stage hypospadias repair with or without transplantation of prepuce and/or skin flaps |
|  | 54308 | Urethroplasty for second stage hypospadias repair (including urinary diversion); less than 3 cm |
|  | 54312 | Urethroplasty for second stage hypospadias repair (including urinary diversion); greater than 3 cm |
|  | 54316 | Urethroplasty for second stage hypospadias repair (including urinary diversion) with free skin graft obtained from site other than genitalia |
|  | 54318 | Urethroplasty for third stage hypospadias repair to release penis from scrotum (eg, third stage Cecil repair) |
|  | 54322 | One stage distal hypospadias repair (with or without chordee or circumcision); with simple meatal advancement (eg, Magpi, V-flap) |
|  | 54324 | One stage distal hypospadias repair (with or without chordee or circumcision); with urethroplasty by local skin flaps (eg, flip-flap, prepucial flap) |
|  | 54326 | One stage distal hypospadias repair (with or without chordee or circumcision); with urethroplasty by local skin flaps and mobilization of urethra |
|  | 54328 | One stage distal hypospadias repair (with or without chordee or circumcision); with extensive dissection to correct chordee and urethroplasty with local skin flaps, skin graft patch, and/or island flap |
|  | 54332 | One stage proximal penile or penoscrotal hypospadias repair requiring extensive dissection to correct chordee and urethroplasty by use of skin graft tube and/or island flap |
|  | 54336 | One stage perineal hypospadias repair requiring extensive dissection to correct chordee and urethroplasty by use of skin graft tube and/or island flap |
|  | 54340 | Repair of hypospadias complications (ie, fistula, stricture, diverticula); by closure, incision, or excision, simple |
|  | 54344 | Repair of hypospadias complications (ie, fistula, stricture, diverticula); requiring mobilization of skin flaps and urethroplasty with flap or patch graft |
|  | 54348 | Repair of hypospadias complications (ie, fistula, stricture, diverticula); requiring extensive dissection and urethroplasty with, flap, patch or tubed graft (includes urinary diversion) |
|  | 54352 | Revision of prior hypospadias repair requiring extensive dissection and excision of previously constructed structures including re-release of chordee and reconstruction of urethra and penis by use of local skin as grafts and island flaps and skin brought in as flaps or grafts |
| Posterior urethral valves | 52400 | Cystourethroscopy with incision, fulguration, or resection of congenital posterior urethral valves, or congenital obstructive hypertrophic mucosal folds |
| Vesicoureteral reflux | 50660 | Ureterectomy, total, ectopic ureter, combination abdominal, vaginal and/or perineal approach |
|  | 50780 | Ureteroneocystostomy; anastomosis of single ureter to bladder |
|  | 50782 | Ureteroneocystostomy; anastomosis of duplicated ureter to bladder |
|  | 50783 | Ureteroneocystostomy; with extensive ureteral tailoring |
|  | 50785 | Ureteroneocystostomy; with vesico-psoas hitch or bladder flap |
|  | 50947 | Laparoscopy, surgical; ureteroneocystostomy with cystoscopy and ureteral stent placement |
|  | 50948 | Laparoscopy, surgical; ureteroneocystostomy without cystoscopy and ureteral stent placement |
|  | 52327 | Cystourethroscopy (including ureteral catheterization); with subureteric injection of implant material |
| Ureterocele | 50660 | Ureterectomy, total, ectopic ureter, combination abdominal, vaginal and/or perineal approach |
|  | 51535 | Cystotomy for excision, incision, or repair of ureterocele |
|  | 52300 | Cystourethroscopy; with resection or fulguration of orthotopic ureterocele(s), unilateral or bilateral |
|  | 52301 | Cystourethroscopy; with resection or fulguration of ectopic ureterocele(s), unilateral or bilateral |
| Urethral stricture disease | 51520 | Cystotomy; for simple excision of vesical neck (separate procedure) |
|  | 51800 | Cystoplasty or cystourethroplasty, plastic operation on bladder and/or vesical neck (anterior Y-plasty, vesical fundus resection), any procedure, with or without wedge resection of posterior vesical neck |
|  | 52275 | Cystourethroscopy, with internal urethrotomy; male |
|  | 52276 | Cystourethroscopy with direct vision internal urethrotomy |
|  | 52281 | Cystourethroscopy, with calibration and/or dilation of urethral stricture or stenosis, with or without meatotomy, with or without injection procedure for cystography, male or female |
|  | 52282 | Cystourethroscopy, with insertion of permanent urethral stent |
|  | 52283 | Cystourethroscopy, with steroid injection into stricture |
|  | 52500 | Transurethral resection of bladder neck (separate procedure) |
|  | 52640 | Transurethral resection; of postoperative bladder neck contracture |
|  | 53000 | Urethrotomy or urethrostomy, external (separate procedure); pendulous urethra |
|  | 53010 | Urethrotomy or urethrostomy, external (separate procedure); perineal urethra, external |
|  | 53020 | Meatotomy, cutting of meatus (separate procedure); except infant |
|  | 53400 | Urethroplasty; first stage, for fistula, diverticulum, or stricture (eg, Johannsen type) |
|  | 53405 | Urethroplasty; second stage (formation of urethra), including urinary diversion |
|  | 53410 | Urethroplasty, 1-stage reconstruction of male anterior urethra |
|  | 53415 | Urethroplasty, transpubic or perineal, 1-stage, for reconstruction or repair of prostatic or membranous urethra |
|  | 53420 | Urethroplasty, 2-stage reconstruction or repair of prostatic or membranous urethra; first stage |
|  | 53425 | Urethroplasty, 2-stage reconstruction or repair of prostatic or membranous urethra; second stage |
|  | 53443 | Urethroplasty with tubularization of posterior urethra and/or lower bladder for incontinence (eg, Tenago, Leadbetter procedure) |
|  | 53450 | Urethromeatoplasty, with mucosal advancement |
|  | 53460 | Urethromeatoplasty, with partial excision of distal urethral segment (Richardson type procedure) |
|  | 53600 | Dilation of urethral stricture by passage of sound or urethral dilator, male; initial |
|  | 53601 | Dilation of urethral stricture by passage of sound or urethral dilator, male; subsequent |
|  | 53605 | Dilation of urethral stricture or vesical neck by passage of sound or urethral dilator, male, general or conduction (spinal) anesthesia |
|  | 53620 | Dilation of urethral stricture by passage of filiform and follower, male; initial |
|  | 53621 | Dilation of urethral stricture by passage of filiform and follower, male; subsequent |
| Neurologic disorders | 63200 | Laminectomy, with release of tethered spinal cord, lumbar |
|  | 63700 | Repair of meningocele; less than 5 cm diameter |
|  | 63702 | Repair of meningocele; larger than 5 cm diameter; |
|  | 63704 | Repair of myelomeningocele; less than 5 cm diameter |
|  | 63706 | Repair of myelomeningocele; larger than 5 cm diameter |
|  | 63707 | Repair of dural/cerebrospinal fluid leak, not requiring laminectomy |
|  | 63709 | Repair of dural/cerebrospinal fluid leak or pseudomeningocele, with laminectomy |
